# The Role of Central Serotonin Neurons and 5-HT Heteroreceptor Complexes in the Pathophysiology of Depression: A Historical Perspective and Future Prospects

**DOI:** 10.3390/ijms22041927

**Published:** 2021-02-15

**Authors:** Dasiel O. Borroto-Escuela, Patrizia Ambrogini, Barbara Chruścicka, Maria Lindskog, Minerva Crespo-Ramirez, Juan C. Hernández-Mondragón, Miguel Perez de la Mora, Harriët Schellekens, Kjell Fuxe

**Affiliations:** 1 Department of Neuroscience, Karolinska Institutet, Biomedicum, Lab B0851, Solnavägen 9, 17 177 Stockholm, Sweden; 2 Department of Biomolecular Science, Section of Morphology, Physiology and Environmental Biology, University of Urbino, Campus Scientifico Enrico Mattei, via Ca’ le Suore 2, I-61029 Urbino, Italy; patrizia.ambrogini@uniurb.it; 3 Observatorio Cubano de Neurociencias, Grupo Bohío-Estudio, Zayas 50, 62100 Yaguajay, Cuba; 4 APC Microbiome Ireland, University College Cork, T12K8AF Cork, Ireland; chrusstek@interia.pl (B.C.); h.schellekens@ucc.ie (H.S.); 5 Małopolska Centre of Biotechnology, Jagiellonian University, 30 252 Kraków, Poland; 6 Department of Neuroscience, University of Uppsala, 75 105 Uppsala, Sweden; maria.lindskog@neuro.uu.se; 7 Instituto de Fisiología Celular, Universidad Nacional Autónoma de México, Mexico City 04510, Mexico; mcrespo@ifc.unam.mx (M.C.-R.); jcmondragon@ifc.unam.mx (J.C.H.-M.); mperez@ifc.unam.mx (M.P.d.l.M.); 8 Department of Anatomy and Neuroscience, University College Cork, T12K8AF Cork, Ireland

**Keywords:** G protein-coupled receptors, heteroreceptor complexes, serotonin receptor, oligomerization, oxytocin receptor, depression

## Abstract

Serotonin communication operates mainly in the extracellular space and cerebrospinal fluid (CSF), using volume transmission with serotonin moving from source to target cells (neurons and astroglia) via energy gradients, leading to the diffusion and convection (flow) of serotonin. One emerging concept in depression is that disturbances in the integrative allosteric receptor–receptor interactions in highly vulnerable 5-HT1A heteroreceptor complexes can contribute to causing major depression and become novel targets for the treatment of major depression (MD) and anxiety. For instance, a disruption and/or dysfunction in the 5-HT1A-FGFR1 heteroreceptor complexes in the raphe-hippocampal serotonin neuron systems can contribute to the development of MD. It leads inter alia to reduced neuroplasticity and potential atrophy in the raphe-cortical and raphe-striatal 5-HT pathways and in all its forebrain networks. Reduced 5-HT1A auto-receptor function, increased plasticity and trophic activity in the midbrain raphe 5-HT neurons can develop via agonist activation of allosteric receptor–receptor interactions in the 5-HT1A-FGFR1 heterocomplex. Additionally, the inhibitory allosteric receptor–receptor interactions in the 5-HT1AR-5-HT2AR isoreceptor complex therefore likely have a significant role in modulating mood, involving a reduction of postjunctional 5-HT1AR protomer signaling in the forebrain upon activation of the 5-HT2AR protomer. In addition, oxytocin receptors (OXTRs) play a significant and impressive role in modulating social and cognitive related behaviors like bonding and attachment, reward and motivation. Pathological blunting of the OXTR protomers in 5-HT2AR and especially in 5-HT2CR heteroreceptor complexes can contribute to the development of depression and other types of psychiatric diseases involving disturbances in social behaviors. The 5-HTR heterocomplexes are novel targets for the treatment of MD.

## 1. Introduction

In the seminal thesis by Kjell Fuxe on “Evidence for the existence of monoamine neurons in the central monoamine neurons” in 1965 (Karolinska Institutet) [1], we identified the serotonin brain stem neurons [1,2,3] using the Falck–Hillarp fluorescence method. Ascending serotonin axonal pathways to the telencephalon and diencephalon originated mainly from the midbrain raphe and para-raphe nerve cell bodies identified by their yellowish fluorescence obtained with the Falck–Hillarp method through conversion of serotonin (5-HT) into a fluorophor with a peak emission at 530 nm [4]. The descending serotonin axon projections to the spinal cord gray matter, forming mainly varicose serotonin nerve terminals, originated from serotonin neurons of the raphe and para-raphe regions of the medulla oblongata and pons [1,5]. Varicose serotonin nerve terminal networks were identified all over the gray matter (dorsal, intermediate and ventral horns) of the spinal cord in low to moderate densities. By determination of the distribution of 5-HT immuno-reactivity in the brain, it became possible to obtain a much improved and complete mapping of the widespread 5-HT nerve terminal networks of the brain [6].

In 1967, we obtained indications for the existence of a serotonin reuptake mechanism in the 5-HT nerve cell bodies and dendrites, axons and nerve terminals, opening up the possibility that antidepressants can act by blocking this mechanism [7]. In 1968, involving collaboration with Nobel Laureate Arvid Carlsson, we obtained results suggesting that imipramine can inhibit the serotonin reuptake mechanism in nerve terminals and cell bodies [8,9]. This was the beginning of the development of selective serotonin reuptake inhibitors (SSRIs). In 1977, we found that antidepressant drugs like amitriptyline have affinity for the d-LSD binding sites but not for the high-affinity 5-HT binding sites [10]. Our interpretation was that these results indicated the existence of two types of 5-HT receptors (5-HT1 and 5-HT2 receptors). In 1979, we found that the degree of the blockade of head-twitches in mice produced by the antidepressants was highly correlated with their affinity for 3H-d-LSD binding sites [11]. The interpretation was that the blockade of one type of 5-HT receptor by antidepressant drugs may contribute to their therapeutic effects. Currently, as many as 13 distinct heptahelical, G protein-coupled receptors (GPCRs) and one ligand-gated ion channel have been identified, divided into seven distinct classes (5-HT(1) to 5-HT(7)) on the basis of their structural and operational characteristics [12].

Serotonin communication operates mainly in the extracellular spaces and cerebrospinal fluid (CSF), using volume transmission (VT) communication with serotonin moving from source to target cells (neurons and astroglia) via energy gradients, leading to the diffusion and convection (flow) of serotonin. Serotonin operates via VT in the extrasynaptic “µm” range to activate the high-affinity serotonin receptors located mainly in extrasynaptic regions but also in synapses [13,14,15].

Modulation by serotonin VT of, e.g., glutamate and GABA synapses in the hippocampus involves 5-HT homoreceptor complexes and many different subtypes of 5-HT heteroreceptor complexes located on neurons and/or astroglia, like serotonin 1A receptor (5-HT1A)-fibroblast growth factor receptor 1 (FGFR1) [16,17,18,19], serotonin 1a receptor (5-HT1A)- serotonin 2A receptor (5-HT2A) [20], serotonin 1A receptor (5-HT1A)- galanin receptor 1 (GalR1) [21,22,23,24], serotonin 1A receptor (5-HT1A)- galanin receptor 1 (GalR1)- galanin receptor 2 (GalR2) [25], serotonin 1A receptor (5-HT1A)- G-protein coupled receptor 39 (GPR39) [26], serotonin 2C receptor (5-HT2C)- growth hormone secretagogue receptor 1A (GHS-R1a) [27,28,29], serotonin 2A receptor (5-HT2A)- oxytocin receptor (OXTR) [30] and serotonin 2C receptor (5-HT2C)- oxytocin receptor (OXTR) [31] heteroreceptor complexes. One emerging concept in depression is that disturbances in allosteric receptor–receptor interactions in highly vulnerable 5-HT1A heteroreceptor complexes can contribute to causing depression and become novel targets for the treatment of depression and anxiety [19,32,33]. Moreover, the 5-HT2A and 5-HT2C receptors are steadily receiving more attention as a therapeutic target for mood disorders [34], and both the GHS-R1a OXTRs have also been implicated in anxiety [35,36].

### New Insights into Understanding Integration of Signals in Heteroreceptor Complexes in the Serotonin Neurons and Its Target Neurons and Their Relevance for Depression

From the literature, we know there exist a large number of 5-HT receptor subtypes, some of which should be activated by agonists to produce antidepressant effects like postjunctional 5-HT1A and 5-HT4 agonists, while others should be blocked to improve depression, like serotonin 5-HT2A, 5-HT2C and 5-HT7 auto-receptors [37,38,39,40]. However, only few articles have been published on the possible role of distinct 5-HT heteroreceptor complexes in depression [19].

Now we introduce a novel hypothesis that a few of these 5-HTR heterocomplexes can contribute to depression by changing their densities and/or allosteric receptor–receptor interactions in the plasma membrane in this disease [32]. Thus, they can be highly vulnerable, with altered function, and become novel targets for the treatment of depression. Vulnerability means changes in the heterocomplex density (up or down) and/or in the allosteric receptor–receptor interactions (increasing or decreasing in strength), leading to dysfunction. The pharmacological analysis of the vulnerable heterocomplexes in models of major depression given receptor protomer ligands and interface-interfering peptides can determine the direction of the current antidepressant drug approach to these vulnerable heterocomplexes. The balance between different 5-HT1A and other 5-HT heteroreceptor complexes can also become altered in depression, contributing to a novel signaling panorama linked to depression development.

The hypothesis goes beyond the serotonin hypothesis of depression and moves into molecular integration performed in diverse 5-HT1A and other 5-HTR heterocomplexes where, e.g., fibroblast growth factor receptor 1 (FGFR1) [19] and oxytocin receptor [30] protomers participate. Thus, if one or more of these receptor protomers exist among the highly vulnerable complexes, they can become novel targets for antidepressant drugs.

Treatment-resistant major depression can be observed with current neuropsychopharmacology. Additionally, SSRIs have had only moderate success, with a need for other treatments like electroconvulsive therapy or transcranial stimulation. It may be related to the problem of recovering normal molecular integration in highly vulnerable 5-HT1A or other 5-HT heterocomplexes with current drugs. However, it is proposed that subchronic treatment with the SSRI fluoxetine and/or acute treatment with ketamine may, in part, reverse the marked changes in integration induced in highly vulnerable 5-HT1A or other 5-HT2AR heterocomplexes by facilitating a return of the integrative malfunction of these complexes towards normality. In the current paper, four types of 5-HT heteroreceptor complexes (5-HT1AR-FGFR1, 5-HT1A-5-HT2AR, OXTR-5-HT2A/C) have been selected to give insights into this novel field with a focus on their relevance for depression.

## 2. 5-HT1A-FGFR1 Heteroreceptor Complexes

In 2012, evidence was obtained for the existence of 5-HT1AR-FGFR1 heteroreceptor complexes in the dorsal rat hippocampus, followed by the discovery of their existence in the dorsal raphe and median raphe of the midbrain [16,17,18,19,41] using proximity ligation assays. Thus, it was clear that both 5-HT1A postsynaptic receptors (hippocampus) and auto-receptors (midbrain raphe) could physically interact with FGFR1. Enhanced allosteric receptor–receptor interactions developed, leading to enhanced FGFR1-mediated plasticity which correlated with antidepressant activity, as evaluated in the forced swim test [16]. Later, in 2017, it was found that combined treatment over a period of two days with FGF2 and the 5-HT1AR agonist 8-OH-DPAT, given i.c.v., only increased the density of the 5-HT1AR-FGFR1 complexes in the CA2 region of the pyramidal cell layer of the hippocampus [42]. This finding was of substantial interest since the projections from CA2 plays a critical role in social memory [43].

**Figure 1:**
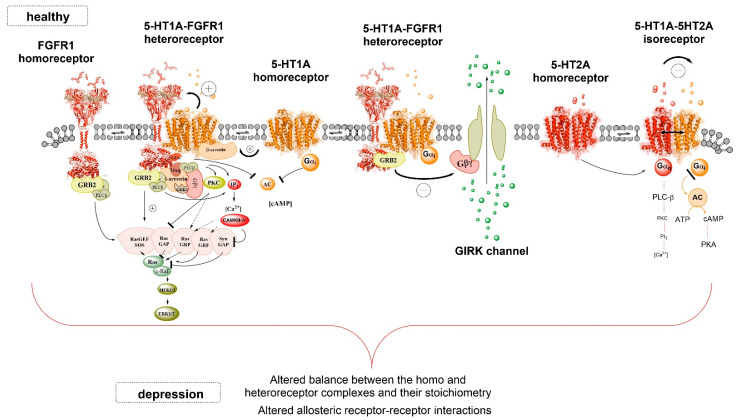
The 5-HT1A-FGFR1 heteroreceptor complexes (to the left) and the 5-HT1A-5-HT2A isoreceptor (to the right) heteroreceptor complexes are illustrated. The term isoreceptor is used for the 5-HT1A-5-HT2A heteromer since the two types of 5-HT receptors are activated by the same transmitter (5-HT). They are in the plasma membrane of control rats (healthy). They are shown to be in balance with the corresponding homoreceptor complexes in the plasma membrane. The FGFR1 homoreceptor complex is presented as a dimer and this is also true also for the 5-HT1A and 5-HT2A homoreceptor complexes. The FGFR1 homoreceptor and the FGFR1 protomer in the heteroreceptor couple are all coupled to ERK1/2, a major signaling pathway for plasticity, via growth factor receptor-bound protein 2 (adaptor protein) and RAS protein. With a curved arrow and plus sign close to it, the allosteric enhancement by the 5-HT1A protomer of the FGFR1 protomer signaling over the GRB2-RAS-ERK1/2 signaling pathway is illustrated. The 5-HT1A receptor protomer is presented as a dimer. As a result, the FGFR1 activation by an FGFR1 agonist is increased, leading to enhanced neuroplasticity involving the ERC1/2 pathway [16]. The 5-HT1A protomer in the heteroreceptor complex mainly signals via Gi/o to inhibit AC activity, like the 5-HT1A homoreceptor, as indicated. The other 5-HT1A-FGFR1 heteromer instead is used to illustrate how the FGFR1 protomer linked to GRB2 upon activation can reduce the coupling of the 5-HT1A protomer to the GIRK channel via an inhibitory allosteric interaction in the heterocomplex. The detailed molecular mechanism is unknown, but the result is an allosteric brake of the 5-HT1AR-induced opening of the GIRK channels. In the dorsal raphe, it leads to an inability of the 5-HT1A auto-receptor to hyperpolarize the dorsal raphe neurons and an increased firing of the dorsal raphe serotonin neurons can develop, leading to antidepressant actions. In the right part of the figure, the 5-HT1A-5-HT2A heteromer is shown as a heterodimer. It is illustrated that the 5-HT2A protomer via an inhibitory allosteric receptor–receptor interaction can reduce its signaling over the Gi/o-AC-PKA pathway. This leads to a dominance of 5-HT2A signaling with depressive effects [20]. The figure especially illustrates how allosteric inhibitory or facilitatory allosteric receptor–receptor interactions among the receptor protomer of the complex can produce antidepressant or depressive events which can help determine if antidepressant or depressive effects develop.

The neurophysiological studies by Ambrogini and colleagues published in Borroto-Escuela (2017) [42] have provided evidence that in Sprague Dawley (SD) rats, the FGFR1 agonist Sun-11602 substantially reduces the 5-HT1A receptor-induced opening of the GIRK channels [44] in the 5-HT1AR-positive pyramidal glutamate nerve cell bodies of the CA1 region of the dorsal hippocampus [42]. The molecular mechanism is likely an antagonistic allosteric interaction in the 5-HT1AR-FGFR1 complex through which the agonist-activated FGFR1 protomer induces a conformational change in the 5-HT1AR protomer, reducing its ability to open GIRK channels (Figure 1). This molecular mechanism is probably also in operation in the 5-HT1A auto-receptor-FGFR1 complex in the midbrain raphe nerve cells [17,18,45]. Evidence for this view has been obtained (Ambrogini et al. 2020, manuscript in prepraration). These results open up new possibilities to develop other rapid antidepressant drugs similar to ketamine [46,47,48], namely, brain-permeable FGFR1 agonists. They also have, besides trophic actions, the ability to rapidly reduce the 5-HT1A auto-receptor signaling in the ascending serotonin neurons from the midbrain. Such drugs should also contribute to the development of rapid antidepressant effects of selective serotonin reuptake inhibitors (SSRIs).

### 2.1. The Flinders Sensitive Line (FSL) Model of Depression

The FSL model is validated and shows depression symptoms like despair and deficits in memory [49]. It is worth mentioning in this respect that whereas SD rats used as a control group showed a reduction of immobility time in the forced swim test upon combined, but not single, i.c.v. treatment over a 48 h period with FGF2 and 8-OH-DPAT, such combined treatment of FSL rats failed to produce any antidepressant effects in this test. Thus, the synergistic effects of the combined FGF2 and 8-OH-DPAT treatment were blocked in FSL rats. However, 8-OH-DPAT only treatment produced a significant reduction of immobility time, indicating antidepressant effects by the 5-HT1A agonist [42]. These antidepressant actions observed in SD rats were associated with significant increases in the density of the 5-HT1AR-FGFR1 complexes in the pyramidal cell layer of the CA2 and CA3 areas and in the dorsal raphe. Thus, differential actions were found in the response to FGFR1 and 5HT1A agonists in the FSL vs. the SD control rats.

The mechanism for such marked differences between both strains is not known but it is proposed that specific conformational changes may have developed in 5-HT1AR and/or the FGFR1 in the FSL strain in comparison to the SD rats, possibly related to a different competing balance with other types of receptors/protein complexes (Figure 1). This biochemical change alters their ability to form single or combined agonist-induced changes in the density of 5-HT1AR-FGFR1 heteroreceptor complexes in the dorsal hippocampus in the FSL strain vs. the SD control rats. It is of great interest that the 5-HT1AR agonist treatment alone can produce antidepressant-like effects only in the FSL rats. The mechanism appears to be linked to its ability in the FSL strain to increase the 5-HT1AR-FGFR1 heteroreceptor complexes in the CA2 and CA3 regions of the dorsal hippocampus, which is not found in controls [42]. The decline in the formation of hippocampal 5-HT1AR-FGFR1 complexes in FSL rats upon combined treatment with FGF2 and the 5-HT1A agonist may also be related to increased competition with the induced formation of FGFR2-5-HT1A, FGFR3-5-HT1A and FGFR4-5-HT1A complexes in the hippocampus.

### 2.2. A Novel Hypothesis on Depression

A disruption and/or dysfunction in the 5-HT1A-FGFR1 heteroreceptor complexes in the raphe-hippocampal serotonin neuron systems can contribute to the development of major depression. It leads to reduced neuroplasticity and potential atrophy in the raphe-cortical and raphe-striatal 5-HT pathways and in all its forebrain networks [19]. It also leads to failure to recruit the raphe 5-HT1A auto-receptors to these complexes in which they may change their function away from hyperpolarization and reduction of neuronal activity by reducing their ability to open the GIRK channels [42]. These events may further lead to abnormal reductions of firing rate in the ascending serotonin neurons and reduce 5-HT synthesis and release. Therefore, 5-HT1A-FGFR1 heteroreceptor complexes may offer a target for novel antidepressants involving hetero-bivalent drugs and multi-targeted drugs, with potential for both acute and long-term actions. Additionally, 5-HT1A auto-receptor-FGFR1 heteroreceptor complexes exist in midbrain serotonin raphe cells [17,18]. Synergistic allosteric receptor–receptor interactions, including agonist coactivation, may contribute to acute antidepressant actions by inter alia recruiting increased numbers of 5-HT1A auto-receptors to form 5-HT1A auto-receptor-FGFR1 complexes. Reduced 5-HT1A auto-receptor function and increased plasticity and trophic activity in the midbrain raphe 5-HT neurons can develop via agonist activation of allosteric receptor–receptor interactions in this receptor complex. The resulting counteraction of atrophy in the raphe-hippocampal serotonin neurons together with FGFR1-induced reduction of the opening of the GIRK channels by the 5-HT1AR auto-receptor could be a major development.

## 3. 5-HT1AR-5-HT2AR Isoreceptor Complexes

Over several decades, we have become aware that among the seven serotonin receptor subtypes, some of them should be blocked, like 5-HT2AR and 5-HT7R, to reduce major depression, while others should be activated, like the postjunctional 5-HT1AR and 5-HT4R [10,11,13,37,38,39,50,51]. There exists clear evidence that antagonism of 5-HT2AR improves the therapeutic actions of SSRIs in major depression [52]. Activation of postjunctional 5-HT1AR in the limbic system is an important action in the mediation of the antidepressant effects of SSRIs. Here, they inhibit the neuronal firing of the limbic networks by increasing serotonin VT which enhances the activation of the postjunctional Gi/o-coupled 5-HT1AR. This leads to opening of the G protein-coupled inwardly-rectifying potassium channels (GIRK, causing hyperpolarization and inhibition of neuronal firing. These findings have relevance since the limbic networks are hyperactive in major depression [39].

The delay seen in the therapeutic effects of SSRIs is likely caused by tolerance development in the 5-HT1A auto-receptor of the midbrain raphe neurons at the cell body/dendritic level in response to the long-term increase in extracellular serotonin levels [37,51,53]. It may involve an increased brake on the opening of the GIRK channel via increased activity in the FGFR1 protomer in the 5-HT1A auto-receptor-FGFR1 heteroreceptor complex of the dorsal and median raphe nerve cells [17,18,42]. The activation of the FGFR1 protomer can be the result of a facilitatory allosteric receptor–receptor interactions in the 5-HT1A auto-receptor-FGFR1 complex upon prolonged activation by serotonin of the 5-HT1A auto-receptor (Figure 1). It is presently unknown whether or not 5-HT1A auto-receptor-5-HT2AR complexes do actually exist in the midbrain raphe nuclei.

The inhibitory allosteric receptor–receptor interactions in the 5-HT1AR-5-HT2AR isoreceptor complex therefore likely have a significant role in modulating mood, involving a reduction of postjunctional 5-HT1AR protomer signaling in the forebrain upon activation of the 5-HT2AR protomer (Figure 1). Such an action should favor depression development. It will be important to test the impact of this receptor complex in modulating depression, as evaluated in the forced swim test with the use of interface-interfering peptides with the ability to break up this isoreceptor complex. Triplet amino acid homologies participate in the receptor interface of heteromeric complexes according to the triplet puzzle theory [54]. They have also been demonstrated in 5-HT1AR-5-HT2AR complexes [20]. Peptides with such triplet homologies should therefore represent potential interface-interfering peptides to be used in these experiments.

The 5-HT1AR-5-HT2AR complex also represents a dynamic complex, since a marked disappearance of this complex was found in the pyramidal cell layer of the CA1 and CA2 area 24 h after exposure to the forced swim test [20]. It is possible that stress may block their formation which can involve increased release of glucocorticoids from the adrenals, activating glucocorticoid receptors in the hippocampus. The activated glucocorticoid receptors then alter neuronal gene transcription in the hippocampus. This can lead to the production of a novel panorama of adapter proteins that can bind to various types of receptor complexes in the plasma membrane of the hippocampal neurons. As a result, the balance between the various homo- and heteroreceptor complexes is changed. In the case of the 5-HT1AR-5-HT2AR, complex it can become markedly reduced, e.g., due to its binding of adapter proteins that reduce the affinity of the 5-HT1AR and 5-HT2AR protomers for each other and/or increase their affinity for other types of receptor protomers.

## 4. Oxytocin (OXTR)-5-HT2AR and OXTR-5-HT2CR Heterocomplexes

The oxytocin nerve terminal networks found in many regions of the brain, like the hypothalamus, nucleus acccumbens, hippocampus and cortical areas, are formed from axons originating mainly from oxytocin nerve cells in the paraventricular hypothalamic regions [55] and operate via volume transmission. The oxytocin receptors have a similar distribution pattern, as the oxytocin nerve terminals and are mainly activated by oxytocin through its extracellular diffusion and flow from the surrounding oxytocin-positive nerve terminal networks. There exists a significant degree of co-distribution of the serotonin and oxytocin terminal networks and their 5-HT2A/C and oxytocin receptor systems in the cortical and subcortical regions of the forebrain. Furthermore, oxytocin can produce anxiolytic actions through oxytocin receptors (OXTRs) expressed in serotonin neurons [35,56].

OXTRs play a significant and impressive role in modulating social and cognitive-related behaviors, like bonding and attachment, reward and motivation, as well as fear, anxiety and stress-related responses [57,58]. These modulations of the neuronal networks involve the formation of heteroreceptor complexes with other GPCRs, like 5-HT2AR, 5-HT2CR [30,31] and D2R [59,60]. The dynamic formation of OXTR-5-HT2AR, OXTR-5-HT2CR and OXTR-D2R heteroreceptor complexes, especially in the limbic cortex and nucleus accumbens, makes possible a fundamental integrative process in the plasma membrane for the modulation of social and cognitive behaviors. It develops through allosteric receptor–receptor interactions present in these complexes that dynamically modulate the recognition, signaling and trafficking of the receptor protomers of the abovementioned receptor complex. Changes in the composition, stoichiometry and degree of activation of receptor protomers can markedly alter the allosteric interactions, e.g., from being inhibitory to becoming facilitatory.

The balance of these heteroreceptor complexes depends on their existence in the same neuron and in the same plasma membrane area, like in the same synapse. The formation of corresponding 5-HT2AR and 5-HT2CR homoreceptor complexes as well as 5-HT2AR-5-HT2CR isoreceptor complexes also participates [61,62,63,64]. It should be noted that social reward demands coordinated activity of the oxytocin and serotonin transmitters in the nucleus accumbens [65].

### 4.1. OXTR-5-HT2AR Heteroreceptor Complexes

These complexes were identified in living cells using flow cytometry-based Fluorescence resonance energy transfer (FRET), including confocal microscopy, and in forebrain sections using a proximity ligation assay (PLA) [30]. To date, they have been found in many neurons of the nucleus accumbens shell and core, layers II and III of the cingulate cortex and the CA2 and CA3 areas of the hippocampus. Their location is mainly linked to the plasma membrane of neurons but they are also found intracellularly due to increased trafficking, confirmed in a cellular model. The neuronal location is based on the use of neuronal markers.

It should be noticed that bidirectional antagonistic allosteric receptor–receptor interactions appear to exist in the OXTR-5-HT2AR heterocomplexes, as determined in cellular models [30]. The results are based on studies on 5-HT2AR and OXTR protomer-mediated signaling over Galphaq proteins, causing an increase in second messenger (IP3) production and in intracellular calcium release. It is important to notice that the 5-HT2AR protomer upon its activation had a dominating role which resulted in a stronger attenuation of the OXTR-mediated Galphaq signaling (IP3 and intracellular calcium levels) than of 5-HT2AR mediated Galphaq signaling. The above results are of substantial interest since the dominating role of the 5-HT2AR protomer, a 5-HT receptor known to enhance depression, may do so in part by reducing OXTR protomer signaling, which improves social behaviors and mood, provided that this antagonistic allosteric cross-talk also exists in the brain OXTR heteroreceptor complexes [65,66,67,68]. It is also of interest that in co-transfected (5-HT2AR and OXTR) living cells, the 5-HT2AR antagonist blocking the 5-HT2AR protomer signaling could not restore the OXTR signaling in the receptor complex. Thus, it seems possible that the inhibitory allosteric mechanism, once put in operation through activation of the 5-HT2AR protomer, is no longer dependent on the agonist activation of the 5-HT2AR protomer orthosteric binding site. Thus, there appears to have developed a constitutive activity in the 5-HT2AR protomer that can pass over the receptor interface and maintain allosteric inhibition of the oxytocin receptor protomer. Similar results were obtained when using OXTR antagonists to counteract the OXTR protomer-induced inhibition of 5-HT2AR protomer signaling. The allosteric inhibition of 5-HT2AR, in this case mediated by the OXTR protomer, was again maintained. It remains to be determined if these inhibitory allosteric mechanisms are similar in the brain’s 5-HT2AR-OXTR heteroreceptor complexes. If this is true, the balance in the signaling of the two receptor protomers in this heteroreceptor complex in the forebrain appears to be a relevant factor for the maintenance of intact complex social behaviors.

### 4.2. OXTR-5-HT2CR Heteroreceptor Complexes

Based on flow cytometry FRET and confocal microscopy, OXTR-5-HT2CR heterocomplexes were first demonstrated in vitro in living cells, followed by observations of their existence in the brain using proximity ligation assays [31]. They appeared to have a widespread distribution which is compatible with the OXTR and 5-HTR2C co-expression observed in mouse whole cortex and hippocampus and in human cortex following in silico analysis of the single cell RNA sequencing data available via the transcriptomic explorer in the Allen Brain Atlas (https://celltypes.brain-map.org/rnaseq/mouse_ctx-hip_smart-seq (accessed on 18 February 2021)). To date, PLA-positive OXTR-5-HT2CR heterocomplexes have been found as red clusters in the dorsal hippocampus and in the retro-splenial granular and agranular cortex. High densities of PLA-positive clusters were found in the pyramidal cell layer of the CA1-3 regions located on the pyramidal glutamate neurons, especially in CA3. Scattered GABA interneurons located inter alia in the oriens and radiatum layers were also identified as being PLA positive. Taken together, the results indicate that the OXTR-5-HT2CR heterocomplexes represent a widespread integrative mechanism in the neuronal networks of the forebrain [31].

Using an intracellular calcium mobilization assay and a time-resolved fluorescence-based IP1 accumulation assay, it was possible to observe a significant attenuation of the OXTR protomer-mediated Galphaq signaling by 5-HT2CR protomer activation [31]. As was the case for the 5-HT2AR protomer, in the 5-HT2AR-OXTR heterocomplex, the Galphaq signaling of the 5-HT2CR protomer was also less affected by the activation of the OXTR protomer. Thus, 5-HT2CR had a dominating role and, upon its activation, it markedly reduced the OXTR protomer signaling in the cellular model used. Such a blunting of signaling in partner receptor protomers forming a heterocomplex with 5-HT2CR has also been observed in the GHSR1a-5-HT2CR heterocomplex [29] as well as in 5-HT2AR-5-HT2CR and 5-HT2BR-5-HT2CR isoreceptor complexes linked to a disappearance of agonist binding to the 5-HT2AR and 5-HT2BR protomers [69]. Moreover, unlike in the case of 5-HTR2AR complexes, the restoration of OTR-mediated signaling following pharmacological 5-HTR2C blockade confirms the hypothesis that homo- and heterocomplexes of 5-HTR2C can be regulated and even disrupted by its antagonists but not agonists [70].

It is also of importance that these neurochemical results demonstrating strong antagonistic allosteric receptor–receptor interactions induced by the 5-HT2CR protomers in the above complexes can explain the behavioral results obtained [31]. Indeed, it was observed that the brain-penetrating 5-HT2CR antagonist SB 242,084 could significantly enhance oxytocin-induced hypoactivity in rodents [36,71] which does not represent an additive action [31]. In fact, when given alone, the 5-HT2C antagonist increased locomotor activity. These results support the view that the blunting of the OXTR protomer signaling by 5-HT2CR protomer activation influences centrally regulated processes with behavioral consequences. Nevertheless, it is highly important to further study the allosteric receptor–receptor interactions in the brain of 5-HT2R heteroreceptor complexes. It seems likely that pathological blunting of the OXTR protomers in 5-HT2AR and especially in 5-HT2CR heteroreceptor complexes can contribute to the development of depression and other types of psychiatric diseases involving disturbances in social behaviors.

## 5. Implications for a Role of 5-HT Heteroreceptor Complexes in Other Types of Brain Disease Besides Depression

The 5-HT nerve terminal networks are also known to participate in, e.g., schizophrenia, drug addiction, anxiety and Parkinson’s disease [72,73]. As for schizophrenia, it is of great interest that serotonin and glutamate interactions have been demonstrated in preclinical models of schizophrenia that, at least in part, take place in 5-HT2AR-mGluR2 heterocomplexes, which affect trafficking and localization involving mouse frontal cortex pyramidal neurons [74,75]. The demonstration of 5-HT2AR-D2R heterocomplexes is also of great relevance for schizophrenia [76,77,78,79], since D2R and 5-HT2AR antagonists represent major drugs in the treatment of schizophrenia. It should be noted that in 2010, a review presented electrophysiological evidence for serotonin–dopamine interactions [73].

Cunningham and colleagues provided strong indications for the existence of 5-HT2AR-5-HT2CR heterocomplexes in the mouse medial prefrontal cortex [80] and could validate in cell cultures a very close interaction between 5-HT2AR and 5-HT2CR [61]. Previously, this group had found an imbalance between 5-HT2A and 5-HT2C receptors in the medial prefrontal cortex, which was linked to motor impulsivity. Synergistic interactions have also been observed in the case of CB1R and 5-HT2CR, which can prevent status epilepticus induced by pilocarpine in rats, which may involve the formation of a heteroreceptor complex [81]. This recent work illustrates that multiple 5-HT heteroreceptor complexes can participate in the modulation of several neuropsychiatric diseases. Their location in the neuronal networks forming the various brain circuits will determine in which diseases they will be implicated.

## 6. Conclusions

The presented research on 5-HT heteroreceptor complexes in the brain indicates that they are molecular centers in the plasma membrane for the dynamic integration of biological signals like neurotransmitters. Disturbances in these integrative processes likely have a significant role in the patho-physiological events that lead to the development of MD. Restoration of these integrative molecular mechanisms should therefore lead to antidepressant actions and the development of novel drugs to treat depression. Such drugs can also lead to an improvement of the slow antidepressant effects of SSRIs and the rapid antidepressant effects of ketamine. The future of treatment of major depression looks promising.
